# Strategic planning for degrowth: What, who, how 

**DOI:** 10.1177/14730952241258693

**Published:** 2024-05-31

**Authors:** Federico Savini

**Affiliations:** Department of Geography, Planning and International Development Studies, 368197 University of Amsterdam, Amsterdam, Netherlands

**Keywords:** city-regions, degrowth, strategic planning, institutions, prefiguration

## Abstract

Degrowth is gaining traction as a viable alternative to mainstream approaches to sustainability. However, translating degrowth insights into concrete strategies of collective action remains a challenge. To address this challenge, this paper develops a degrowth perspective for strategic spatial planning as well as a strategic approach for degrowth. I argue that a degrowth transition needs to address three strategic issues: depth, agency, and trajectory. Degrowth strategies aim for satiation, the satisfaction of all essential needs in a particular society. To do so, they rely on diffused societal power, raising from existing practices of reduction. Strategies also follow a nonlinear trajectory that seeks to prefigure satiation, popularize it among the masses, and then pressure existing institutions. Strategic spatial planning offers important insights for dealing with these challenges but needs to embrace satiation as a strategic goal. It can do so by creating complementarities between prefigurative practices that perform satiation. The article defines and illustrates these processes by looking at the making of Amsterdam’s ‘doughnut’ strategy.

## Introduction: The strategic challenge of degrowth

This paper develops a degrowth perspective for strategic spatial planning, contributing to both strategic thought in degrowth research and degrowth research in planning theory. These two emerging fields, I argue, can benefit from each other in identifying strategies for dealing with contemporary socio-ecological urgencies.

Degrowth envisions a *planned reduction* of economies’ material throughput, starting with less-necessary forms of production and consumption (D’Alisa et al., 2014; Schmelzer et al., 2022). It couples this downscaling with a *planned improvement* of services that deliver wellbeing without leaving a significant ecological footprint; the initial recipients would be social groups that do not enjoy high basic standards of living (Büchs & Koch, 2019; Hickel, 2020). A degrowth agenda pursues an economy that thrives on care, culture, education, health, and ecological regeneration (Jackson, 2009). A transition to this new economy, degrowth advocates argue, could be achieved through democratic processes that are autonomous from the socio-cultural and political institutions that sustain the growth imperative (Asara et al., 2013).

Despite increasing interest, degrowth ‘has neither support from a comprehensive coalition of social forces nor any consent to its agenda among the broader population’ (Buch-Hansen, 2018:157). To build this consent, degrowth scholars – particularly degrowth economists – have indicated numerous regulations and measures that can be used to realize degrowth (Fitzpatrick et al., 2022, 2022; Sekulova et al., 2013). Though many are anticapitalist, others coexist with current sustainability policies (Mastini et al., 2021). Many of these proposals target the use of land, highlighting modes of housing and mobility that dovetail with social justice and planetary boundaries (see for example Nelson & Schneider, 2018; Savini et al., 2022; De Castro Mazarro et al., 2023; Kębłowski, 2023; Xue & Kębłowski, 2022, more later). Although no country has yet adopted something approximating a degrowth agenda (Fanning et al., 2022), some cities have begun questioning how growth contributes to sustainability and collective wellbeing and they have undertaken some key degrowth interventions.^1^ Examples include the promotion of slow mobility, housing cooperatives, shared living, moratoria on evictions, caps on rents or emissions, targets on phasing out fossil fuels in Amsterdam, Glasgow, Barcelona, and Copenhagen.

There are examples of feasible degrowth policies. What degrowth research currently lacks, then, is not policy proposals but insights into strategies that can publicly legitimize those policies. Although it has just begun to tackle this problem systematically (Barlow et al., 2022), in practice ‘debates and controversies over strategies employed within each source of the degrowth movement have been most intense’ (Demaria et al, 2013:207). In this debate, *strategic indeterminacy* – the avoidance of a unitary strategy or political direction – is considered both a barrier to degrowth and a source of its potential (Escobar, 2018). At one level, the degrowth community tries to include any initiative that downscales the demand for materials and strives for social equality. At another, it aspires to ‘greater coordination, planning, and prioritization of strategic mixes within specific contexts’ (Herbert et al, 2021). Overall, today’s strategic challenge for degrowth is to establish whether and how popular consensus can be built around a multitude of actions aiming to downscale economies’ material throughput.

This paper addresses this strategic challenge by combining degrowth strategic thinking with a degrowth critique of strategic planning. First, the paper unpacks the *what*, *who,* and *how* of degrowth strategic planning. I define these as *depth*, *agency,* and *trajectory* respectively. My argument is that a strategic process for a degrowth agenda is organized around satiation as a driving principle; it caters to a diffuse agency; and it follows a nonlinear path that combines prefiguration, popularization, and pressure.

In the second part of the article, I build on these insights to argue that strategic planning theory can play a pivotal role in advancing a degrowth agenda. The reason is that strategic planning has historically catered for diffuse agency and nonlinearity in socio-spatial transformations. Yet, to embrace degrowth it must take satiation as a guiding principle for a strategic vision of reduction of both unnecessary consumption and production. I then define two processes that a degrowth strategic planning needs to consider to achieve these goals: *synergy* and *regionalization*.

I will illustrate these two processes by looking at the making of the (so-called) Municipality of Amsterdam’s ‘doughnut strategy’. This is a strategic framework explicitly oriented to satiation and approved by the council of the city. This brief example shows synergy and regionalization as constitutive processes of a strategic planning trajectory that aims at reducing the urban economy’s impact on the ecosystem and increasing wellbeing. In conclusions, I will reflect on the limits of this practice from a degrowth perspective and on the role of planners in this process.

## Challenges to a degrowth strategy: Depth, agency, and trajectory

Degrowth incites politics to imagine the socio-political transformation that would reduce and slow down the social metabolism, namely the total amount of materials, water and energy being used in society (Savini, 2023b). Within degrowth research, it is generally agreed that degrowth needs to combine both radical and reformist approaches to social transitions and to embrace some sort of flexible strategic pluralism to not alienate the many voices that appeal to reduction (see Chertkovskaya, 2022, building on Wright, 2010). While stressing the need for a planned process of reduction, degrowth economists seem to envision a role of the state, complemented with strong direct democracy, but hardly explain how this process would come about, what strategies could trigger a degrowth planning trajectory and which actors should drive it (see for example Durand et al., 2024). The (scarce) degrowth scholarship directly concerned with planning currently overlooks the strategic challenge of fostering widespread public support, a prerequisite for pursuing planned degrowth as a mainstream political agenda.

To address this gap, it is necessary to first identify what are the actual strategic issues at stake building on the literature that has conceptualized the notion of degrowth. In what follows, I build on that literature to distinguish between the *depth*, *agency*, and *trajectories*, that are peculiar to degrowth, and pinpoint the degrowth transition’s strategic uniqueness.

## Depth (what): Satiation

Degrowth is commonly understood as a radical project of socio-cultural transformation. To question the degrowth transition’s *depth* is to identify the social norms targeted by degrowth strategic action.

Degrowth has been largely (even if not always explicitly) associated with post- or anticapitalism. The growth economy is rooted in enclosures of the commons, colonialism, and the exploitation of labor (D’Alisa et al., 2014; Feola, 2020; Schmelzer et al., 2022; Schmid, 2019). Because capitalism requires incessant surplus accumulation – hence its exploitation of labor and nature – degrowth is generally understood as anticapitalist (Vandeventer et al., 2019). Yet there is no consensus about what specifically a postcapitalist degrowth strategy should imply. Schmidt, for example, argues that postgrowth must target institutions that keep individuals and organizations dependent on competition, profit, and financial transactions (Schmid, 2023). Feola argues that degrowth is anticapitalist because it unmakes capitalist institutions (Feola, 2019). Others identify private property (van Griethuysen, 2010) or the exploitation of socially reproductive labor (Barca, 2019) as the key institution. Anarchist approaches to degrowth focus instead on hierarchy and the state (Trainer, 2019).

The first strategic challenge is therefore to identify the actual strategic target of a degrowth agenda. Bellamy Foster (2011) has argued that degrowth (particularly of the Latouchian stamp) does not sufficiently question imperialism and class inequalities. Degrowth, he claims, should be understood as ecosocialism. In response, Kallis has argued that a transition to socialism hardly ensures the downscaling of material throughput. In fact, ‘growth can continue in so far as the workers refrain from consuming all of the product for the satisfaction of their immediate human needs, and instead invest it to increase production, to satisfy their higher needs of tomorrow’ (Kallis 2015: x). As Saito echoed, ecosocialism ‘does not exclude the possibility of pursuing further sustainable economic growth once capitalist production is overcome’ (Saito, 2023: 209) and he advances the notion of degrowth communism. In sum, the recognition of the environmental limits to growth is distinctive trait of a postgrowth alternative but this does not necessarily translate into one single political theory (Wiedmann et al., 2020).

Degrowth envisions a shift in the social norms that sustain the compulsion to grow, accumulate, exploit, and prioritize productivity (it rejects economism too). In their place, it calls for social relations to revolve around the principle of *satiation* (Bilancini & D’Alessandro, 2012; Pettini & Musikanski, 2022). Satiation, a core principle of degrowth, refers to a society meeting all its essential needs. This focus on satiation promotes reducing excess production and consumption. As a social value, satiation shapes how people interact with each other and their environment, replacing competition and exploitation with symbiosis, acceptance, and stability (Rosa, 2019; Buch-Hansen & Nesterova, 2023). By prioritizing satiation as a political goal, degrowth seeks liberation from the compulsion to consume unnecessarily. This pursuit opens pathways for different socio-political structures, urging degrowth scholars (and activists) to explore context-specific means to achieve a socio-political transition.

As a social norm, satiation can affect social relations only insofar as individuals see it as legitimate (Goldmann, 2005). Because it is seen as legitimate, it can produce new institutions and regulations (e.g. resource caps or moratoria). For this reason – and others that I explain later – degrowthers have strongly advocated for forms of prefiguration that nurture degrowth values (Trainer, 2019). As Soper puts it (2023), degrowth is primarily a transition towards an ‘alternative hedonism’: a form of self-fulfillment that rejects pure utilitarianism in which voluntary limitation and simplicity are motivated by immaterial pleasure, happiness, and satisfaction. Satiation thus connects a larger universe of degrowth values, such as care for others and nature (Muraca, 2012). The degrowth imaginary’s anticapitalism derives from its rejection of accumulation and acceleration as logics of social organization – the very logics through which capital exists. Satiation become, in turn, thresholds beyond which excess, overconsumption, luxury, profit, and accumulation (of land, assets, housing, etc.) can be identified as ‘unacceptable’ or ‘immoral’ against the backdrop of human consumption transgressing planetary boundaries (Gough, 2017).

## Agency (who): Diffuse agency

The second strategic problem for degrowth is that of *agency*. Degrowth advocates generally agree that degrowth is grounded in grassroots cultures, particularly groups struggling for public transport, housing, healthy food, care, equal rights, public space, and land rights, and against extractivism (Demaria et al., 2013; Latouche, 2009). Degrowth literature tends to be critical of existing governmental institutions. Yet, paradoxically its policy proposals tend to identify the state as potentially initiating a degrowth transition (Alami et al., 2023). ‘Contemporary degrowth advocacy hence suffers from a “tension” between viewing the state as incapable of initiating transformational change and making a political appeal to it to do precisely this’ (Koch, 2022: 7). When reflecting on political agency, proponents of degrowth suggest that degrowth needs a new social movement, one that bridges economic and ecological concerns, building an ‘ecological class consciousness’ (Barca, 2019).

Within degrowth research, this understanding of agency is rooted in Gramsci’s critique of the state. The state is not reduced to a government or particular actor but grasped as a system of social norms and institutions that define the boundaries of agency itself. In this theory of the integral state, the hegemony of growth is manifest in the widely held belief that the interests of the dominant class (those that accumulate for profit) are universal to all social groups. For degrowth, this hegemony is why people struggle to reject the idea that profits must expand if essential services and good standards of living are to be provided (i.e. trickledown economics). This is particularly important for planners, many of whom see urban development – often through permissive real-estate investment strategies – as a precondition for urban equality (Gunder, 2010).

From this perspective, a degrowth transition may rather require a partnership between political organizations, civic organizations and grassroots movements that prefigure degrowth futures (D’Alisa et al., 2013; D’Alisa & Kallis, 2020). This approach to radical agency stems from the autonomist tradition, Gramscian Marxism, and Castoriadis’s work on the autonomous production of social imaginaries. Degrowth entails interrupting and negating the growth machine – exploitation, profit, and the reinvestment of surplus. It refuses capitalist values such as work, compulsive consumption, individual competition, and profit maximization.

According to this framework, agency is *diffused* (or distributed) because it is *practiced* in everyday choices to repair, share, divest, gift, and regenerate peoples and nature. These everyday practices of prefiguration, which exist in the current economy’s interstices, are the pillars upon which a degrowth strategy can be built. In sum, a strategy emerges from cracks in the current system (Buch-Hansen, 2018; Koch, 2019). Degrowth actors are ‘signifying agents’ producing alternative, contentious meanings that counteract mainstream sustainability practices (Demaria et al., 2013). A degrowth transition is built from the practice (of reduction). It has no single initiator.

Practices of degrowth emerge where and when the contradictions of growth-dependent economies are most visible. These moments include the sensation of burnout and poor quality of life (Meissner, 2019); dissatisfaction with urban consumerism (Alexander, 2014); recognition of technological fixes’ inability to decouple GDP from environmental degradation (Haberl et al., 2020); consciousness of eco-efficiency’s rebound effects of (Robra et al., 2020); liberation from housing debt (Bohnenberger, 2021); and pleasure in walking and cycling.

## Trajectory (how): Prefiguration, popularization, and pressure

Degrowth literature has hardly explored the *trajectories* along which strategies emerge and effect change. Degrowth research still lacks a long-term vision of how change evolves, though it clearly rejects neoclassical Marxism’s linear grammar.

As Meissner explains, degrowth research has attended closely to ‘prefiguration’: the act of performing in the present what the future society can be (Meissner, 2021). Degrowth praises voluntary simplicity, collectives reclaiming the commons, reduced working hours, communal agriculture, and collective, low-impact housing (for example, see Chatterton, 2016; Nelson & Schneider, 2018). Yet the considerable attention given to prefiguration too readily equates the challenge of leading a transition with that of practicing the future. These are different and require different skills and tactics. Prefiguration alone traps strategic debates over degrowth in localism (Xue, 2014). In fact, prefigurative practices constantly risk being marginalized, ridiculed, isolated, and coopted by incumbent powers (Savini & Bertolini, 2019). Though sometimes inspiring, they often lack the scale to instigate a power shift.

In dealing with this strategic issue, Meissner claims that deep cultural change demands that prefiguration be coupled with *popularization* and *pressure* (Meissner, 2021). Popularization involves establishing synergies and linkages among degrowth practices and other practices that may not (yet) identify with that term. Popularization entails inducing increasing numbers of people to accept degrowth by ‘mobilizing existing cultural practices and identities for a degrowth transition’ (Meissner, 2021: 524). This is a cultural process that involves framing existing practices using degrowth vocabulary and building what Mouffe terms ‘left populism’ (Mouffe, 2018). *Pressure* is the creation of degrowth movements that can tackle existing institutions by interrupting perpetual growth. It involves ‘a practice of resistance to a given process or status quo (e.g., deforestation or highway expansion). This resistance can consist of blocking a process (e.g., by climbing a tree that is about to be cut) or in exerting pressure on actors to behave in a certain way’ (Meissner, 2021). Peaceful civil disobedience and the ‘hacking’ of existing juridical frameworks to institute policies for urban commons (Micciarelli, 2022) are examples of pressure practices that are grounded in prefiguration and legitimated by popularization.

A degrowth transition is therefore likely to follow a nonlinear trajectory. It is based on prefiguration, which occurs in the growth economy’s interstices, and is likely to involve activists, progressive public employees, the self-employed, and civic organizations whose daily work adheres to degrowth principles. Degrowth practices thrive on synergies among one another. This increases degrowth’s popular legitimacy, building a political movement that pushes for regulatory change. And that change enables new practices and reinforces this transitional loop.

As the movement organizer maree brown has shown, strategy is ‘emergent’ because it is ‘performance based’ (see also Hillier, 2008) and builds on ‘a multiplicity of relatively simple interactions’ (Obolensky, 2014). An emergent strategy is not planned from the top but resembles the synergies that connect flocks of birds, networks of mushrooms, and swarms of bees and other insects. Motivated by specific, simple purposes, these units create ‘critical connections rather than critical mass’ (maree brown, 2017). A strategy emerging from prefiguration is built up of such connections, like a mosaic (Treu et al., 2020).

This understanding makes it possible to overcome the divides between those advocating grassroots movements and those promoting the state. It also puts controversies between radicalism and reformism, which often fragment social movements, into perspective. In short, strategic reflection would foster multiple trajectories, enacted in synergy to achieve social change. Degrowth strategies can refuse to comply with certain rules and interrupt the pursuit of growth. They can hack existing processes by reframing them as degrowth. They can demand the removal of existing rules, whether through divestment, moratoria, or ending subsidiaries for fossil fuels. Or they can reinterpret existing rules and practices in an ecological fashion, much as housing cooperatives do when tweaking social housing regulations, for example.^2^

## A degrowth critique of strategic planning

My review of the literature on strategy and degrowth has advanced three key arguments. First, a degrowth transition aims to *institute* a principle of satiation. Degrowth requires a planning process that is geared to build public pressure around an agenda of reduction of both (unnecessary) production and consumption. Second, degrowth entails diffuse agencies. It should not be led by one single (group of) actors. Third, it builds on prefiguration, striving to popularize degrowth and in so doing begin contesting existing institutions and building new ones. My argument is that strategic spatial planning can contribute significantly to dealing with these three challenges because the distance between strategic planning scholarship and degrowth research on strategy is not as large as it might seem. As it will be elaborated below, the reason is that strategic planning was – in its original and most radical conception – devised as a practice to build legitimacy around new social norms and institutions, to support diffuse agency, and to trigger nonlinear pathways to transformation.

In the early 1990s, planning’s so-called ‘strategic turn’ (Salet & Faludi, 2000) emerged in reaction to the socio-ecological damage wrought by urban growth. Rather than focusing on producing operational plans, this turn was a response to the ‘void’ created by the erosion of the public planning capacities that had founded welfare states and provided safety nets below the socio-spatial inequalities produced by growth (Hajer, 2003). This ‘void’ was not a lack of plans. It was instead a lack of vision of the long-term transformation of a territory, and the lack of an agreed understanding among urban actors of the social, ecological, and economic impact of spatial transformations in city-regional spaces (Healey, 2004). At a historical moment when public agencies were becoming more fragmented across governance networks, strategic planners were preoccupied with finding ways to direct socio-spatial transformation without a unitary plan of socio-spatial change (Healey, 1998, 2007). Before they turned to design and implementation, radical strategic planners produced alternative visions of socially inclusive, ecological, *and* economically prosperous futures. Often, these visions included multiple instances of resistance against the rampant commodification of space (Albrechts, 2004).

Radical strategic planning became a practice to institute procedures, arenas and instruments that could cater for social and environmental justice. To do so, it shifted the attention from operational plans to exploratory processes resulting in long-term visions (Balducci, 2011). Against the push for pure market-led land transformation, strategic planning strove to multiply possibilities for socio-spatial change by creating complementarity between levels of planning action, from the everyday practices of spatial use to governmental policymaking (Hillier, 2011). Strategic planning sought to *liberate* planning from the technical prescriptions underpinning developmentalist, linear, and functionalist approaches to urban transformation. Radical strategic planning, writes Albrechts, provided ‘direction without destination, movement without prediction,’ seeking to ‘tackle problems, raise awareness, meet challenges, and broaden the scope of the possible’ (Albrechts, 2015: 513). It aimed to encourage ‘hopes and dreams, appeal to values (equity, social justice), provide a frame for decisions, and challenge existing knowledge, conventional wisdom, and practices’ (idem).

Despite its different realizations worldwide, strategic planning thought was built on the evidence that strategies where needed to reach deep, diffuse, and non-linear transformations, just as degrowth does today. Strategic planners aimed at enlarging the range of voices dealing with spatial problems, to widen the space for politics (Mäntysalo et al, 2011). This meant to resist the constant pressure of a neoliberal ideology that pushed to straitjacket planning into a technocratic procedure (Raco & Savini, 2019). In fact, strategic planning has often failed to maintain its depth by disengaging with the most radical voices. It reproduced established ideologies (Metzger et al, 2021). In so doing, we could argue, it ended to be strategic, and planners lost their role of orchestrators of visionary processes (see for example Harrison et al, 2021).

These problems of contemporary strategic spatial planning may require a new visionary impulse, one that is suitable to deal with the multiple ongoing social, ecological, and economic crisis and their spatial causes. The absence of degrowth ideas in strategic planning is therefore surprising given the potential of degrowth to inform these radical socio-ecological visions. Although issues of the transformation and use of land are key to the degrowth critique (Demaria et al., 2019, Savini, 2021; Kaika et al., 2023; Krähmer, 2022), planners’ voices remain marginal in the degrowth debate (examples are Barry, 2019; Ferreira & von Schönfeld, 2020; Hackworth, 2018; Rydin, 2013; Savini et al., 2022; Schmid, 2023; Xue, 2021). The works I have just cited are a wake-up call, an admonition to reject dominant growth imperatives in planning in view of current socio-ecological challenges. They urge that the regulatory conditions under which socio-spatial change occurs should be adapted to meet the challenge of planetary overshoot and socio-ecological injustice. These voices propose redesigning planning procedures, tools, and modes of regulation (Durrant et al., 2023), starting with those that regulate how value is captured through development. These planners are also aware that localism can trap initiatives, claiming that planning must also look at regional and national scales (Xue & Kębłowski, 2022).

These are clear steps toward a planning theory (and practice) of degrowth. Yet it is also evident that these changes will not occur automatically thanks to there being some enlightened planners, for most planners’ worldview and professional ethic remain wedded to urban growth (Lamker & Schulze Dieckhoff, 2022). As Davoudi argues, the response to this lack of vision should be prefigurative, not instrumental: the performance of the future through planning practices that ‘negate’ prevailing growth assumptions (Davoudi, 2023). Like degrowthers, planners have started recognizing that change requires prefiguration. Yet planning also needs to define strategies for *reproducing the values* mobilized in those prefigurative practices and protecting them from the constant threat of marginalization (see also Savini, 2023a). In sum, planning for degrowth needs to address its strategic position before rethinking its regulatory toolkit. To do so, it needs to put the goals of reduction of production and consumption next to its goals of socio-ecological justice.

Current strategic planning theory and practice seldom address reduction. It also rarely envisions divestment, interruption, slowdown, and refusal as ways of addressing growing urban footprints (globally) and social injustice amid a rapidly worsening climate crisis. As I have argued elsewhere, this lacuna results from planning’s historical concern with economic redistribution rather than ecological stability (Savini, 2021). Strategic planning sought, and is still searching for, inclusive and green growth. It sees itself as a balancing – not proactive – force that addresses the side effects of unregulated economic expansion, be they sanitary, social, aesthetic, or environmental (Hirt & Campbell, 2023).

Within planning, the insurgent tradition has been especially vocal about refusal, reduction, and divestment (Miraftab, 2009; Uitermark & Nicholls, 2017). These works are concerned to give voice to those dispossessed and exploited in the pursuit of economic growth, processes that intersect closely with racism, gendered oppression, etc. Much like degrowth literature, insurgent planning recognized the need to adopt nonanthropocentric worldviews and question established hegemonies (Sandercock, 2022). It clearly advocates for prefiguration, celebrating the power of grassroots initiatives. Yet it neither coalesced into a program of reduction and satiation nor theorized the processes that leads to popularization and pressure for political and institutional change.

Conversely, strategic planning theories concerned with ecology have adapted to mainstream sustainability policies that pursue efficiency and greening of existing economies. They repeatedly omit the impact of, for example, electrification on hinterlands and exploited labor; the forms of land use required to produce renewable energy (eg. lithium or coltan mines); and the displacement of emissions from cities to global hinterlands (eg. through deindustrialization and landfill). As Ferreira puts it, these planning approaches became dependent on linear innovation and foreclosed radical socio-spatial imaginaries (2020). As Xue argues (2022), in planning, the ideology of growth has been shielded from questioning by planners’ drive to negotiate various interests (or simply modesty). This hinders the emergence of new imaginaries (see also Shepherd et al., 2020).

Orienting strategic planning toward degrowth may help address this lack of imagination. On one side, the literature mentioned above shows that strategic planning has dealt with the problem of how to trigger transformative policies under conditions of diffuse agency. However, it has not questioned limitless economic expansion (Xue, 2021; Ruiz-Alejos & Prats, 2022). On another, degrowth strategic thought recognizes the problem of satiation but does not explain how to move beyond prefiguration toward institutional change. In sum, ‘degrowth scholarship paid relatively little attention to identifying the types of institutional policies and practices that could support broader change that goes beyond small-scale localized practices’ (Kaika et al., 2023: 12). In what follows, I will conceptualize two processes of strategic planning that can address this challenge.

## Synergy and regionalization: The foundation of strategic planning for degrowth

If agency is diffuse, if the strategic target is a long-term vision of reduction, and if the trajectory of change is emergent, then a degrowth strategy inevitably should begin by identifying the political subjects and its boundaries of action. To do so, this strategy needs to build on prefiguration, because it is there that the first image of a degrowth future is delineated. Yet, it must move beyond that, and turn those prefigurative practices into an organized form of agency able to pressure institutions. Just as the strategic challenge of the early 1990s was that of creating regional imaginaries able to boost the role of city-regional politics within global markets, today it is that of defining a geopolitical imaginary for a degrowth polity able to create institutions that counteract the global growth imperative. The role of spatial planners is essential to reach this strategic goal.

A polity is ‘a metaphorical space that demarcates the ‘political sphere’ from other spheres’ (Palonen, 2003: 179). It is thus a boundary of action within which political issues can be raised and addressed. For degrowth, these issues include excess and wealth inequality, environmental damage, neocolonial exploitation, minimum standards of living, and forms of consumption and production that destroy environments but contribute little to collective wellbeing. These issues have no one, universally applicable solution for they are relative to specific geographical scales. Low-income groups in the Netherlands, for example, certainly have a bigger environmental footprint than waste pickers in India. Yet their footprint is five times lower than the top 10% of earners in Europe (Chancel, 2022).

Although environmental economists attempt to address these questions (Millward-Hopkins et al., 2020), they overlook how satiation and excess are politically constructed in socio-spatial contexts. In addressing this limitation, degrowth has largely contested extractivism and advocated for social equality, progressive taxation, moratoria, and divestments from fossil fuels at the global or national scales. Recently, they have targeted the European polity (Mastini et al., 2021; Otchere-Darko, 2023). These attempts have not translated into territorially bound strategic visions.

The tradition of strategic planning, by contrast, shows how the production of geographical boundaries is a precondition for collective action. Spatial planning, in other words, occurs as process of defining common understandings of place among involved actors. According to Ostrom’s theory of institutional change, these boundaries are produced when actors question and coproduce collective social norms (Kiser & Ostrom, 2000; see also Savini, 2018). They define boundaries while identifying common challenges. As Healey puts it, ‘struggles and debates about places and their qualities thus provide a theatre of experience through which people get drawn into thinking about what it means to be a member of a political community, to have a public voice, acknowledge differences and to work out what they might value ‘in common’ about their surroundings’ (2018: 66).

Under the hegemony of a growth ideology in planning, it is certainly hard to imagine the spontaneous emergence of a planning process oriented to reduction. Yet, degrowth strategic planning can (and should) build on the energy of prefiguration to popularize degrowth and pressure politics. This process can be understood as *synergy*: the creation of interdependencies and complementarities among existing degrowth practices, and between those practices and existing institutions.

Synergy occurs when civic practices and governments recognize that they are either ‘mutually supportive’ or ‘embedded’ in each other (Evans, 1996)^3^ and this creates the condition for coproduction (Ostrom, 1996). This coproduction, fostered by planners, can create a powerful movement for change. However, for this process to succeed, governments need to value these alternative practices as drivers of transformation. In sum, in a condition of diffuse agency, for a deep shift in values, strategic planning begins by recognizing that a multitude of prefigurative practices of reduction are legitimate makers of the future that need to cooperate. Planners can be the stewards of this cooperation.

Establishing synergies never occurs in a geographical vacuum. As dense systems of socio-spatial relations, synergies need concrete spaces in which to prosper. The concept of *regionalization* is useful in understanding the relationship between synergy and space. In Giddens’ structuration theory, regionalization is the process through which social relations reproduce sites, the physical locations at which social norms are reflexively performed (Giddens, 2013).^4^ A region is, therefore, a relational space, produced by the routinized performance of social norms through dense practices. It is in these spaces that social norms are contested and negotiated (Urry, 2014). The synergy among degrowth practices thus thrives in spaces where satiation voluntary simplicity, interruption, and commoning are performed. For degrowth, regions are strategic because practices are densest at this scale (Giddens, 2013: 122). Further, they provide both common ground on which to build shared meaning and enough distance to avoid initiatives being locked into localism.

Synergy and regionalization require a large repertoire of planning tools and processes able to create a common frame of reference, establish a vocabulary, identify a shared problem, and recognize that satiation is a multifaceted challenge. The actors concerned with synergy and regionalization first try to grasp the commonalities among existing practices by reflecting critically on how they complement or follow from each other. Planners can do so by recognizing that the reduction of something (e.g., aviation) means the increase of something else (e.g., clean air, high-quality housing, health, recreation spaces). As I will show in the example below, synergy and regionalization occur when public planners and the agents of prefiguration start recognizing their complementarities and interdependencies, and do so while defining their contribution to a city-regional future that embraces reduction.

## An example of synergy and regionalization: Amsterdam’s doughnut

To pursue synergy and regionalization, degrowth planners face one main challenge: creating and mobilizing tools that convey the idea that less is more or, in other words, that the reduction of production and consumption opens spaces for health, care, wellbeing, and prosperity. The task is to popularize the message that limiting unnecessary consumption and production promises liberation from the biophysical, economic, ideological, and financial constraints of economic growth, thus improving ecological and social conditions. To date, no planning strategy has unequivocally embraced such a narrative, although there are many degrowth-inspired social practices that contest the current system of compulsive consumption and profit.

The following example shows an attempt to popularize an agenda of reduction, building on prefiguration and diffuse agency, to create a planning vision of the Amsterdam city-region. Due to these goals (e.g., prioritizing reduction), the strategic process aimed for a deep social value shift, placing reduction at its core and building wider consensus around it. However, the heterogeneous network of actors, with no single dominant force, meant a linear policy-to-implementation approach was not feasible.

It is generally known as the ‘doughnut strategy’ of Amsterdam, because, in its making, planners have used the model developed by Kate Raworth in her book (2017) and her work as a consultant for city governments. The ‘doughnut’ is a metaphor for a model of economic development that rejects the possibility of infinite growth. According to this framework, the economy is enclosed by an ecological ceiling – the conditions necessary for humanity’s survival on the planet – and a social foundation – the minimum living standards required for a thriving human life. When applied to policy making, the doughnut model suggests that (governments) need to, on the one hand, reduce the forms of consumption and production that favor ecological overshoot (i.e. fossil fuels) and, on the other, increase the provision of services to those social groups that do not meet basic living standards (i.e. health care or access to education). It couples ecological reduction with social justice targets.

In 2018, the municipality of Amsterdam (led by Marieke van Dornick, the newly appointed, green-left alderwoman in spatial planning) explicitly adopted this framework to combine the goals of sustainability and social justice in one urban strategy.^5^ Although this strategy has not reduced flows of materials (Municipality of Amsterdam, 2020),^6^ the doughnut metaphor triggered synergetic and regionalizing processes that permeate the city’s political discussions on sustainability. Amsterdam set the objective of halving its consumption of primary abiotic materials by 2030, a decrease of 2.3 billion kg each year (for historical background see Savini, 2019). For planners, to adopt this framework meant to take the perspective of satiation for new regulations and land use policy. At the same time, they needed to create public pressure towards this goal. To do so, they had to survey and connect the multiplicity of prefigurative practices in the city that already embraced this goal, catering to a diffuse agency. They built synergies among them and took the city as the boundary within which to do so. This process, reportedly, popularized the idea of reduction and reportedly created the conditions to pressure the municipality to reach reduction targets. This process can be traced by looking at three key processes that underpinned the ‘doughnut strategy’ between 2018 and 2019.

Amsterdam’s spatial planning department and a consultancy firm (Circle Economy) sponsored a process of identifying commonalities and conflicts among the city’s ongoing sustainable development projects. Four workshops were held to establish whether and how hundreds of projects were contributing toward meeting ecological and social targets. The ecological indicators chosen included CO_2_ emissions, raw materials input to stock, and ocean acidification. The social indicators included physical and mental health, loneliness, mental stress, and access to housing and healthy food. The result of this process was Amsterdam’s so-called circular strategy (Municipality of Amsterdam, 2019), which linked existing projects and identified the conditions for their success. It also revealed that improvements in the city’s economic indicators between 2012 and 2018 (e.g. employment) came at the cost of worsening social-psychological indicators (e.g. loneliness) (Municipality of Amsterdam, 2020).

The legacy of Amsterdam’s doughnut strategy includes self-organized processes of synergy and regionalization. In 2019, an NGO active in one of the city’s poorer neighborhoods (ie. Zuidoost) initiated the so-called ‘Donut deals.’ These were intended to create synergies among municipal offices, civic organizations, and businesses to help poorer social groups while simultaneously developing ways of reusing and reducing materials. The first deal involved schools developing repair skills with reused materials, a self-managed biogas facility, and citizens learning how to install thermal insulation to their homes. Nine deals have been signed to date. They combine social and ecological targets, even if they do not identify with degrowth.

The doughnut metaphor stimulated the emergence of the so-called ‘doughnut coalition (*Donut Coalitie*), a synergy among 400 projects in the Amsterdam city-region that share a common understanding of sustainability as reducing ecological overshoot and increasing wellbeing. It includes NGOs, firms, social projects, and professionals engaged in urban gardening and sharing mobility, and encompasses issues from feminism to plastic reuse. The coalition is more symbolic than political: it reflects a diverse landscape of practices that recognize the idea of limits as central to their work. It also regionalizes an existing network of actors within the Amsterdam city-region.

In 2022, the doughnut strategy was included as one of the key choices taken in the city’s Comprehensive Vision Amsterdam 2050 (Omgevingsvisie, 2050; City of Amsterdam, 2022). In seeking to orient Amsterdam’s future development, this strategic document encompasses transportation, green space, health, housing, renewable energy, and water, among other things. Its fourth strategic pillar recognizes that urban growth (understood as growth in jobs and houses) cannot continue as it is and that economic expansion must be in the sectors of care, sport, housing commons, participation, and education.

As these examples demonstrate, synergy unfolded through the planners’ engagement with the multiplicity of prefigurative practices already occurring in the city. This engagement was built through stable networks of support between public officials, civic and market organizations. It made the doughnut metaphor popular in the public debate. This process was possible, however, by taking the city-region as the boundary of interaction and coproduction, what I understand as regionalization.

It is important to stress that these processes have clear limitations. Many of the projects touched on above overlook the relations between consumption and growth, power, and techno-fixes (Calisto Friant et al., 2023). Moreover, the initiatives I have described scarcely question major polluters, developers’ profit-seeking strategies, or spatial concentrations of excessive wealth. At the moment, these initiatives seem to frame the problem of growth as being the responsibility of urban dwellers, who should better manage their daily consumption. The doughnut trajectory neither considers the politics of environmental (in)justice nor attempts a dialog with especially radical socio-ecological grassroots movements. These are major shortcomings in terms of socio-ecological justice, which might compromise this policy's long-term viability.

Here, I did not intend to celebrate the initiative undertaken in Amsterdam but to unpack how synergy and regionalization work in practice. By engaging with prefiguration, planners can always exclude the most radical voices, focusing on areas that do not present socio-ecological challenges, and pursue synergy and regionalization in a superficial way. If this happens, strategic planning will inevitably empty degrowth of its radical potential and make it ineffective in achieving either social or ecological targets.

## Conclusions: A degrowth turn in strategic spatial planning

Degrowth research lacks a strategic planning theory. To address this limitation, this paper unpacked the strategic problems of degrowth. It argued for a renewed theory of strategic spatial planning, one that embraces satiation as the central guiding principle. So far, strategic spatial planning theory has been silent within degrowth research and practice, while it has historically developed some of the conceptual (and practical) tools to contribute to it. Engaging with degrowth will enrich strategic planning theory as well, allowing it better to respond to worsening socio-ecological conditions. In this article, I have tried to complement these two fields of research and practice. In so doing, I have aimed to advance a scholarship in degrowth planning that has only just begun to emerge.

To address the strategic challenge of degrowth, I first identified the depth, agency, and trajectory of a degrowth transition. As shown in the first part of the paper, degrowth research has already begun dealing with issues of cultural hegemony, the state and the relation between prefiguration and political pressure. In sum, the peculiarity of a degrowth transition is that it is premised on social norms of satiation, which call unnecessary production and consumption into question. Moreover, this transition is not led by a single group of actors; agency is diffuse because it counters a system that concentrates power. Finally, this transition builds on yet transcends prefiguration. Through nonlinear trajectories, degrowth strives to be popularized and then channeled into pressure exerted on existing institutions that favor growth over ecological regeneration and justice.

Planners faced similar conditions in the early 1990s, the heyday of strategic planning practice. Radical strategic planners actively pursued coproduction under conditions of diffuse agency. They also understood that strategies must be open-ended and appreciate nonlinearity. However, the planning tools and procedures developed over the last decades have not foregrounded satiation. Strategic planning, I argue, can benefit from a *degrowth turn* to revive its radical potential and envision a path toward socio-spatial change that is compatible with ecological limits. Building on this awareness, I have conceptualized the processes of synergy and regionalization as two steps of a degrowth planning strategy. Synergy occurs when complementarity and embeddedness among prefigurative practices are recognized. Regionalization localizes that synergy into spaces that become reference points for strategic action.

The concepts of synergy and regionalization are already familiar in strategic planning theory. They stress that, to trigger strategic processes, it is essential for planners to grasp the existing landscape of prefigurative practices, directly engage with them, connect them through frames of meaningful interaction, and define a common understanding of the territory in which those practices coexist. In this paper, I argued that strategic spatial planning needs to go back to these two foundational processes to be able to address the extreme urgency of today’s social and ecological challenges (i.e., ecological breakdown and its related socio-political implications). Yet, it needs to do so by focusing on those practices that see reduction as imperative. In cities, these practices are increasingly common. Examples include housing cooperatives, ecological social housing, squats, community agriculture, food sovereignty, collective voluntary simplicity, and networks of care, education, and health.

The article has illustrated how these processes in the making of the so-called Amsterdam Doughnut strategy. The building of synergies and regionalization from a myriad of prefigurative practices within the city constituted the first step towards an agenda that recognized limits to economic growth and placed satiation at its core. The example showed how strategic planning unfolded under conditions of diffuse agency, with a combination of grassroots and publicly driven initiatives. Spatial planners mobilized existent prefigurative practices, connected them around a shared idea of a prosperous city, and made them popular within urban policy arenas, which in turn facilitated the creation of networks supporting new prefigurative practices; this process was eventually oriented towards a city-wide spatial strategy.

Synergy and regionalization do not happen in a vacuum but need to be actively pursued by degrowth-minded spatial planners. These planners can be embedded in both governmental and civic organizations but will have to (constantly) avoid the risk of cooptation by pro-growth discourses that may hide neoliberal tendencies behind the claim of reduction. Degrowth research (and practice) suggests, however, that by maintaining satiation as the central goal, by cherishing diffuse agency, and by connecting prefiguration to popularization and pressure, it is possible to reduce this risk. Synergy among practices that pursue the reduction of production and consumption is a precondition to avoid this risk. Furthermore, these synergies must be regionalized into spaces that respond to both ecological and social imperatives. In so doing, the city-region can find a new centrality as a space for degrowth.

## Notes

1. Most cities adopt the term postgrowth, which, put briefly, refers to policy approaches that reject the assumption that states’ or cities’ economic growth is *necessary* to meet social and climate targets. Degrowth is a postgrowth approach, but it specifically problematizes the politics of reduction, tackles excess, and promotes sufficiency to downscale the economy in a socially just way.

2. These trajectories are taken from Mahoney and Thelen’s framework of institutional change (2010).

3. Evans does not use the term prefiguration, but that of civic practices.

4. Giddens also stresses the importance of routinization: the reiterative use of jargon, words, ways of doing things and ways of saying that tends to reproduce social norms and favor their structuration into institutions.

5. Marieke van Dornick was the first woman ever to have responsibility for spatial planning in Amsterdam. Moreover, she is the first and hitherto only legislator to combine land, sustainability, and planning under one unique councillorship.

6. Annual data can be found on Amsterdam’s circular monitor platform.

## References

[bibr1-14730952241258693] Alami I Copley J Moraitis A (2023) The ‘wicked trinity’ of late capitalism: Governing in an era of stagnation, surplus humanity, and environmental breakdown. Geoforum 1: 103691.

[bibr2-14730952241258693] Albrechts L (2004) Strategic (spatial) planning reexamined. Environment and Planning B 31: 743–758.

[bibr3-14730952241258693] Albrechts L (2015) Ingredients for a More Radical Strategic Spatial Planning. Environment and Planning B: Planning and Design 42(3): 510–525.

[bibr4-14730952241258693] Alexander S (2014) Simplicity. In: D’Alisa G Demaria F Kallis G . (ed) Degrowth. Routledge, pp.161–164.

[bibr5-14730952241258693] Asara V Profumi E Kallis G (2013) Degrowth, democracy and autonomy. Environmental Values 22(2): 217–239.

[bibr6-14730952241258693] Balducci A (2011) Strategic planning as exploration. Town Planning Review 82(5): 529–546.

[bibr7-14730952241258693] Barca S (2019) The Labor(s) of Degrowth. Capitalism Nature Socialism 30(2): 207–216.

[bibr8-14730952241258693] Barlow N Regen L Cadiou N Chertkovskaya E Hollweg M Plank C Schulken M Wolf V (2022) Degrowth & strategy: How to bring about social-ecological transformation. MayFly.

[bibr9-14730952241258693] Barry J (2019) Planning in and for a post-growth and post-carbon economy. In: Davoudi S Cowell R White I Blanco H (ed) The Routledge Companion to Environmental Planning. Routledge, pp.120–129.

[bibr10-14730952241258693] Bilancini E D’Alessandro S (2012) Long-run welfare under externalities in consumption, leisure, and production: A case for happy degrowth vs. Unhappy growth. The Economics of Degrowth 84: 194–205.

[bibr11-14730952241258693] Bohnenberger K (2021) Can ‘Sufficiency’ reconcile social and environmental goals? A Q-methodological analysis of German housing policy. Journal of Housing and the Built Environment 36(1): 171–189.

[bibr12-14730952241258693] Buch-Hansen H (2018) The Prerequisites for a Degrowth Paradigm Shift: Insights from Critical Political Economy. Ecological Economics 146: 157–163.

[bibr13-14730952241258693] Buch-Hansen H Nesterova I (2023) Less and more: Conceptualising degrowth transformations. Ecological Economics 205: 107731.

[bibr14-14730952241258693] Büchs M Koch M (2019) Challenges for the degrowth transition: The debate about wellbeing. Futures 105: 155–165.

[bibr15-14730952241258693] Calisto Friant M Reid K Boesler P Vermeulen WJ Salomone R (2023) Sustainable circular cities? Analysing urban circular economy policies in Amsterdam, Glasgow, and Copenhagen. Local Environment 28: 1331–1369.

[bibr16-14730952241258693] Chancel L (2022) Global carbon inequality over 1990–2019. Nature Sustainability 5(11): 931-938.

[bibr17-14730952241258693] Chatterton P (2016) Building transitions to post‐capitalist urban commons. Transactions of the Institute of British Geographers 41(4): 403–415.

[bibr18-14730952241258693] Chertkovskaya E (2022) A strategic canvas for degrowth: In dialogue with Erik Olin Wright. In: Barlow N Regen L Cadiou N Chertkovskaya E Hollweg M Plank C Wolf V (eds) Degrowth & Strategy: How to bring about social-ecological transformation. MayFly, pp.56–71.

[bibr19-14730952241258693] D’Alisa G Demaria F Cattaneo C (2013) Civil and Uncivil Actors for a Degrowth Society. Journal of Civil Society 9(2): 212–224.

[bibr20-14730952241258693] D’Alisa G Demaria F Kallis G (2014) Degrowth: A vocabulary for a new era. Routledge.

[bibr21-14730952241258693] D’Alisa G Kallis G (2020) Degrowth and the State. Ecological Economics 169: 106486.

[bibr22-14730952241258693] Davoudi S (2023) Prefigurative planning: Performing concrete utopias in the here and now. European Planning Studies, online first.

[bibr115-14730952241258693] De Castro Mazarro A Kaliaden GR Wende W , et al. (2023) Beyond urban ecomodernism: How can degrowth-aligned spatial practices enhance urban sustainability transformations. Urban Studies 60(7): 1304–1315.

[bibr23-14730952241258693] Demaria F Kallis G Bakker K (2019) Geographies of degrowth: Nowtopias, resurgences and the decolonization of imaginaries and places. Environment and Planning E: Nature and Space 2(3): 431–450.

[bibr24-14730952241258693] Demaria F Schneider F Sekulova F Martinez-Alier J (2013) What is degrowth? From an activist slogan to a social movement. Environmental Values 22(2): 191–215.

[bibr25-14730952241258693] Durand C Hofferberth E Schmelzer M (2024) Planning beyond growth: The case for economic democracy within ecological limits. Journal of Cleaner Production 437, 140351.

[bibr26-14730952241258693] Durrant D Lamker C Rydin Y (2023) The Potential of Post-Growth Planning: Re-Tooling the Planning Profession for Moving beyond Growth. Planning Theory & Practice 0(0): 1–9.

[bibr27-14730952241258693] Escobar A (2018) Designs for the pluriverse: Radical interdependence, autonomy, and the making of worlds. Duke University Press.

[bibr28-14730952241258693] Evans P (1996) Government action, social capital and development: Reviewing the evidence on synergy. World Development 24(6): 1119–1132.

[bibr29-14730952241258693] Fanning AL O’Neill DW Hickel J Roux N (2022) The social shortfall and ecological overshoot of nations. Nature Sustainability 5(1): 26-36.

[bibr30-14730952241258693] Feola G (2019) Degrowth and the Unmaking of Capitalism. ACME: An International Journal for Critical Geographies 18(4): 977–997.

[bibr31-14730952241258693] Feola G (2020) Capitalism in sustainability transitions research: Time for a critical turn? Environmental Innovation and Societal Transitions 35: 241–250.

[bibr32-14730952241258693] Ferreira A von Schönfeld KC (2020) Interlacing planning and degrowth scholarship: A manifesto for an interdisciplinary alliance. DisP-The Planning Review 56(1): 53–64.

[bibr33-14730952241258693] Ferreira A von Schönfeld KC Tan W Papa E (2020) Maladaptive planning and the pro-innovation bias: Considering the case of automated vehicles. Urban Science 4(3): 41.

[bibr34-14730952241258693] Fitzpatrick N Parrique T Cosme I (2022) Exploring degrowth policy proposals: A systematic mapping with thematic synthesis. Journal of Cleaner Production 1: 132764.

[bibr35-14730952241258693] Foster JB (2011) Capitalism and Degrowth: An Impossibility Theorem. Monthly Review, January 1. Available at: https://monthlyreview.org/2011/01/01/capitalism-and-degrowth-an-impossibility-theorem/

[bibr36-14730952241258693] Giddens A (2013) The constitution of society: Outline of the theory of structuration. John Wiley & Sons.

[bibr37-14730952241258693] Goldmann K (2005) Appropriateness and consequences: The logic of neo‐institutionalism. Governance 18(1): 35–52.

[bibr38-14730952241258693] Gough I (2017) Heat, greed and human need: Climate change, capitalism and sustainable wellbeing. Edward Elgar Publishing.

[bibr39-14730952241258693] Gunder M (2010) Planning as the ideology of (neoliberal) space. Planning Theory 9(4): 298–314.

[bibr40-14730952241258693] Haberl H Wiedenhofer D Virág D Kalt G Plank B Brockway P Fishman T Hausknost D Krausmann F Leon-Gruchalski B Mayer A Pichler M Schaffartzik A Sousa T Streeck J Creutzig F (2020) A systematic review of the evidence on decoupling of GDP, resource use and GHG emissions, part II: Synthesizing the insights. Environmental Research Letters 15(6): 065003.

[bibr41-14730952241258693] Hackworth J (2018) Urbanization, planning and the possibility of being post-growth. In: Ward K Jonas AEG Miller B Wilson D (eds) The Routledge Handbook on Spaces of Urban Politics. Routledge, pp.197–205.

[bibr42-14730952241258693] Hajer M (2003) Policy without polity? Policy analysis and the institutional void. Policy Sciences 36(2): 175–195.

[bibr43-14730952241258693] Harrison J Galland D Tewdwr-Jones M (2021) Regional planning is dead: Long live planning regional futures. Regional Studies 55(1): 6–18.

[bibr44-14730952241258693] Healey P (1998) Building institutional capacity through collaborative approaches to urban planning. Environment and Planning A 30(9): 1531–1546.

[bibr45-14730952241258693] Healey P (2004) The treatment of space and place in the new strategic spatial planning in Europe. International Journal of Urban and Regional Research 28(1): 45–67.

[bibr46-14730952241258693] Healey P (2007) Urban complexity and spatial strategies: Towards a relational planning for our times. Taylor & Francis, vol 14.

[bibr47-14730952241258693] Healey P (2018) Creating public value through caring for place. Policy & Politics 46(1): 65–79.

[bibr48-14730952241258693] Herbert J Barlow N Smessaert J Bardi C (2021) Degrowth strategies: thinking with and beyond Erik Olin Wright. Degrowth.info Resilience, October 25, 2021.

[bibr50-14730952241258693] Hickel J (2020) Less is more: How degrowth will save the world. Random House.

[bibr51-14730952241258693] Hillier J (2008) Plan(e) Speaking: A Multiplanar Theory of Spatial Planning. Planning Theory 7(1): 24–50.

[bibr52-14730952241258693] Hillier J (2011) Strategic navigation across multiple planes: Towards a Deleuzean-inspired methodology for strategic spatial planning. Town Planning Review 82(5): 503–527.

[bibr53-14730952241258693] Hirt SA Campbell SD (2023) The Planner’s Pentangle: A Proposal for a Twenty-First-Century Model of Planning. Journal of Planning Education and Research, Online first.

[bibr54-14730952241258693] Jackson T (2009) Prosperity without growth: Economics for a finite planet. Routledge.

[bibr55-14730952241258693] Kaika M Varvarousis A Demaria F March H (2023) Urbanizing degrowth: Five steps towards a Radical Spatial Degrowth Agenda for planning in the face of climate emergency. Urban Studies 60(7):1191-1211.

[bibr56-14730952241258693] Kallis G (2015) Can there be green socialist growth? A commentary on John Bellamy Foster. In: Entitle. Available at: https://entitleblogdotorg3.wordpress.com/2015/11/03/can-there-begreensocialist-growth-a-commentary-on-john-bellamy-foster-part-ii/ (accessed 2 June 2023).

[bibr116-14730952241258693] Kębłowski W (2023) Degrowth is coming to town: What can it learn from critical perspectives on urban transport? Urban Studies 60(7): 1249–1265.

[bibr57-14730952241258693] Kiser LL Ostrom E (2000) The three worlds of action: A metatheoretical synthesis of institutional approaches. Polycentric Games and Institutions 1: 56–88.

[bibr58-14730952241258693] Koch M (2019) The state in the transformation to a sustainable postgrowth economy. Environmental Politics 29(1):115-133.

[bibr59-14730952241258693] Koch M (2022) State-civil society relations in Gramsci, Poulantzas and Bourdieu: Strategic implications for the degrowth movement. Ecological Economics 193: 107275.

[bibr60-14730952241258693] Krähmer K (2022) Degrowth and the city: Multiscalar strategies for the socio-ecological transformation of space and place. City 26(2–3): 316–345.

[bibr61-14730952241258693] Lamker CW Dieckhoff VS (2022) Becoming a post-growth planner: Inner obstacles to changing roles. In: Savini F Ferreira A von Schönfeld KC (eds) Post-Growth Planning: Cities Beyond the Market Economy. Routledge, pp.189-202.

[bibr62-14730952241258693] Latouche S (2009) Farewell to growth. Polity.

[bibr63-14730952241258693] Mäntysalo R Balducci A Kangasoja J (2011) Planning as agonistic communication in a trading zone: Re-examining Lindblom’s partisan mutual adjustment. Planning Theory 10(3): 257–272.

[bibr65-14730952241258693] Mastini R Kallis G Hickel J (2021) A green new deal without growth? Ecological Economics 179: 106832.

[bibr66-14730952241258693] Meissner M (2019) Against accumulation: Lifestyle minimalism, de-growth and the present post-ecological condition. Journal of Cultural Economy 12(3): 185–200.

[bibr67-14730952241258693] Meissner M (2021) Towards a cultural politics of degrowth: Prefiguration, popularization and pressure. Journal of Political Ecology 28(1): 511–532.

[bibr68-14730952241258693] Metzger J Allmendinger P Kornberger M (2021) Ideology in practice: The career of sustainability as an ideological concept in strategic urban planning. International Planning Studies 26(3): 302–320.

[bibr69-14730952241258693] Micciarelli G (2022) Hacking the legal. In: Savini F Ferreira A von Schönfeld KC (eds) Post-Growth Planning: Cities Beyond the Market Economy. Routledge, pp.112-126.

[bibr70-14730952241258693] Millward-Hopkins J Steinberger JK Rao ND Oswald Y (2020) Providing decent living with minimum energy: A global scenario. Global Environmental Change 65: 102168.

[bibr71-14730952241258693] Miraftab F (2009) Insurgent planning: Situating radical planning in the global south. Planning Theory 8(1): 32–50.

[bibr72-14730952241258693] Mouffe C (2018) For a left populism. London: Verso.

[bibr73-14730952241258693] Municipality of Amsterdam (2019) Amsterdam circular 2024, strategic vision, city of Amsterdam. Report for the Municipality of Amsterdam. Accessed on 2 June 2023. https://assets.amsterdam.nl/publish/pages/867635/amsterdam-circular-2020-2025_strategy.pdf

[bibr74-14730952241258693] Municipality of Amsterdam (2020) Circular Monitor. Report for the Municipality of Amsterdam. Accessed on 12 May 2023. https://assets.amsterdam.nl/publish/pages/867635/amsterdam_circular_monitor.pdf

[bibr75-14730952241258693] Muraca B (2012) Towards a fair degrowth-society: Justice and the right to a ‘good life’ beyond growth. Futures 44(6): 535–545.

[bibr76-14730952241258693] Nelson A Schneider F (2018) Housing for Degrowth: Principles, Models, Challenges and Opportunities. Routledge.

[bibr77-14730952241258693] Obolensky MN (2014) Complex adaptive leadership: Embracing paradox and uncertainty. Gower Publishing, Ltd.

[bibr78-14730952241258693] Ostrom E (1996) Crossing the great divide: Coproduction, synergy, and development. World Development 24(6): 1073–1087.

[bibr79-14730952241258693] Otchere-Darko W (2023) Scaling-up degrowth: Re-imagining institutional responses to climate change. Urban Studies, 60(7): 1316-1325.

[bibr114-14730952241258693] Palonen K (2003) Four times of politics: Policy, polity, politicking, and politicization. Alternatives 28(2): 171–186.

[bibr80-14730952241258693] Pettini A Musikanski L (2022) Doomed to Consume? Non-satiation as a Flaw in the Current Economic Paradigm and What Communities Can Do About It. International Journal of Community Well-Being 6(1): 63-78.

[bibr81-14730952241258693] Raco M Savini F (2019) A New Technocracy? Landscapes of Knowledge in Contemporary Cities. Bristol: Policy Press.

[bibr82-14730952241258693] Raworth K (2017) Doughnut economics: Seven ways to think like a 21st-century economist. Chelsea Green Publishing.

[bibr83-14730952241258693] Robra B Heikkurinen P Nesterova I (2020) Commons-based peer production for degrowth? - The case for eco-sufficiency in economic organisations. Sustainable Futures 2: 100035.

[bibr84-14730952241258693] Rosa H (2019) Resonance: A sociology of our relationship to the world. John Wiley & Sons.

[bibr85-14730952241258693] Ruiz-Alejos C Prats V (2022) In quest of implementing degrowth in local urban planning policies. Local Environment 27(4): 423–439.

[bibr86-14730952241258693] Rydin Y (2013) The future of planning. Policy Press.

[bibr87-14730952241258693] Saito K (2023) Marx in the Anthropocene: Towards the Idea of Degrowth Communism. Cambridge University Press.

[bibr88-14730952241258693] Salet WGM Faludi AKF (2000) Revival of strategic spatial planning. Amsterdam: Elsevier.

[bibr89-14730952241258693] Sandercock L (2022) Once upon a planet: Planning for transition from ego-driven to eco-driven economies. In: Savini F Ferreira A von Schönfeld KC (eds) Post-Growth Planning: Cities Beyond the Market Economy. Routledge, pp.203-215.

[bibr90-14730952241258693] Savini F (2018) Responsibility, polity, value: The (un)changing norms of planning practices. Planning Theory, 18(1), 58–81.

[bibr91-14730952241258693] Savini F (2019) The economy that runs on waste: Accumulation in the circular city. Journal of Environmental Policy & Planning 21(6): 675–691.

[bibr92-14730952241258693] Savini F (2021) Towards an urban degrowth: Habitability, finity and polycentric autonomism. Environment and Planning A: Economy and Space 53(5): 1076–1095.

[bibr93-14730952241258693] Savini F Bertolini L (2019) Urban experimentation as a politics of niches. Environment and Planning A: Economy and Space 51(4): 831–848.

[bibr94-14730952241258693] Savini F (2023a) Maintaining autonomy: Urban degrowth and the commoning of housing. Urban Studies 60(7): 1231–1248.

[bibr95-14730952241258693] Savini F (2023b) Futures of the social metabolism: degrowth, circular economy and the value of waste. Futures. Volume 150, June 2023, 103180.

[bibr96-14730952241258693] Savini F Ferreira A von Schönfeld KC (2022) Post-Growth Planning: Cities Beyond the Market Economy. Routledge.

[bibr97-14730952241258693] Schmelzer M Vetter A Vansintjan A (2022) The future is degrowth: A guide to a world beyond capitalism. Verso Books.

[bibr98-14730952241258693] Schmid B (2019) Degrowth and postcapitalism: Transformative geographies beyond accumulation and growth. Geography Compass 13(11): e12470.

[bibr99-14730952241258693] Schmid B (2023) Post-growth municipalism: Exploring the scalar constitution, strategic relevance, and legal viability of the municipal scale for tackling growth dependencies. Local Environment 1: 1-18.

[bibr100-14730952241258693] Sekulova F Kallis G Rodríguez-Labajos B Schneider F (2013) Degrowth: From theory to practice. Journal of Cleaner Production 38: 1–6.

[bibr101-14730952241258693] Shepherd E Inch A Marshall T (2020) Narratives of power: Bringing ideology to the fore of planning analysis. Planning Theory 19(1): 3–16.

[bibr117-14730952241258693] Soper K (2023) Post-growth living: For an alternative hedonism. Verso Books.

[bibr102-14730952241258693] Trainer T (2019) An Anarchism for Today: The Simpler Way. Capitalism Nature Socialism, 30(4): 87-103.

[bibr103-14730952241258693] Treu N Schmelzer M Burkhart C (2020) Degrowth in movement (s): Exploring pathways for transformation. John Hunt Publishing.

[bibr104-14730952241258693] Uitermark J Nicholls W (2017) Planning for social justice: Strategies, dilemmas, tradeoffs. Planning Theory 16(1): 32–50.

[bibr105-14730952241258693] Urry J (2014) Time and space in Giddens’ social theory. In: Bryant C Jary D (eds) Giddens’ theory of structuration. Routledge, pp.160–175.

[bibr106-14730952241258693] Van Griethuysen P (2010) Why are we growth-addicted? The hard way towards degrowth in the involutionary western development path. Journal of Cleaner Production 18(6): 590–595.

[bibr107-14730952241258693] Vandeventer JS Cattaneo C Zografos C (2019) A Degrowth Transition: Pathways for the Degrowth Niche to Replace the Capitalist-Growth Regime. Ecological Economics 156: 272–286.

[bibr108-14730952241258693] Wiedmann T Lenzen M Keyßer LT Steinberger JK (2020) Scientists’ warning on affluence. Nature Communications, 11(1): 3107. 10.1038/s41467-020-16941-y PMC7305220 32561753

[bibr109-14730952241258693] Wright EO (2010) Envisioning real utopias. Verso Books.

[bibr110-14730952241258693] Xue J (2014) Is eco-village/urban village the future of a degrowth society? An urban planner’s perspective. Ecological Economics 105: 130–138.

[bibr111-14730952241258693] Xue J (2021) Urban planning and degrowth: A missing dialogue. Local Environment, 27(4): 404–422.

[bibr112-14730952241258693] Xue J (2022) A critical realist theory of ideology: Promoting planning as a vanguard of societal transformation. Planning Theory, 21(2): 109–131.

[bibr113-14730952241258693] Xue J Kębłowski W (2022) Spatialising degrowth, degrowing urban planning. Local Environment 27(4): 397–403.

